# Bidirectional Influence of the COVID-19 Pandemic Lockdowns on Health Behaviors and Quality of Life among Chinese Adults

**DOI:** 10.3390/ijerph17155575

**Published:** 2020-08-02

**Authors:** Xiuqiang Wang, Si Man Lei, Shenglong Le, Yanxiang Yang, Boyi Zhang, Wu Yao, Zan Gao, Sulin Cheng

**Affiliations:** 1 Exercise Translational Medicine Center, Shanghai Center for Systems Biomedicine, Shanghai Jiao Tong University, Shanghai 200240, China; wangxiuqiang@sjtu.edu.cn (X.W.); alicelei@um.edu.mo (S.M.L.); longsonlok@sjtu.edu.cn (S.L.); 2 Key Laboratory of Systems Biomedicine (Ministry of Education), Shanghai Center for Systems Biomedicine, Shanghai Jiao Tong University, Shanghai 200240, China; 3 Faculty of Education, University of Macao, Macao, China; 4 Faculty of Sport and Health Sciences, University of Jyväskylä, 40014 Jyväskylä, Finland; 5 Exercise, Health and Technology Center, Department of Physical Education, Shanghai Jiao Tong University, Shanghai 200240, China; yanxiang.yang@tum.de (Y.Y.); zbyboyee@gmail.com (B.Z.); yaowu@sjtu.edu.cn (W.Y.); 6 School of Kinesiology, University of Minnesota, Minneapolis, MN 55455, USA; gaoz@umn.edu

**Keywords:** diet, pandemic, physical activity, sedentary behavior, sleep

## Abstract

Background: The coronavirus disease 2019 (COVID-19) pandemic has created challenges that have caused profound changes in health behaviors. This study aimed to explore how COVID-19 is affecting the health-related quality of life (QoL) among Chinese adults. Methods: The data of health-related behaviors and QoL were collected via online surveys from 2289 adults (mean age = 27.8 ± 12 years) who had been isolated at home for an average of 77 days. Results: More than 50% of the respondents reported that their time engaged in daily physical activity (PA) decreased, while sedentary behavior (SB) time increased compared with that before the lockdown. Only 20% of the respondents reported engaging in moderate-to-vigorous PA, 23% of adults reported changed their diets to be healthier, and 30% reported consuming more vegetables, fruits, and milk products than before home-isolation. During home-isolation, 75.2% of the adults rated their sleep quality as very good, and 65% reported that they were satisfied with their QoL. Sleep quality mediated the relationship between PA and QoL. Conclusion: The two-to-three-month home-isolation has had mixed effects on adult health behaviors in China. The participants were found to have focused more on their eating quality and patterns, which had a positive influence on their QoL. However, people should be encouraged to exercise at home with limited space to maintain a generally healthy lifestyle during a prolonged quarantine.

## 1. Introduction

Since its outbreak in China in December 2019, coronavirus disease 2019 (COVID-19) has infected more than 10.08 million people and has resulted in at least 483,000 deaths worldwide [1]. The pandemic, declared as a global public health emergency by the World Health Organization (WHO) [2], has led many governments and public health agencies to take drastic mitigation measures, including community-wide lockdowns, home quarantines, working-from-home, social distancing, and the prohibition of social gatherings, to stop the spread of COVID-19 and reduce the risk of human-to-human transmission.

The rapid spread of the novel coronavirus along with swift actions taken in response to the COVID-19 pandemic has created a host of new challenges that have brought profound changes and have affected the normal routines of health behaviors and lifestyles for people of all ages, such as restricting outdoor or free-living physical activity (PA), increasing sedentary time, disrupting sleep, and, consequently, adversely affecting the health-related quality of life (QoL) during a nonpandemic era [3]. A recent study showed that the COVID-19 pandemic has negatively affected the mental health (e.g., stress and depression) and QoL among Chinese adults [4]. Before the COVID-19 pandemic, most Chinese people, particularly young adults and those living in large cities, were under various pressures regarding their daily life, such as work-related competition, housing, and daily travel from home to the workplace [5]. However, empirical evidence concerning changes in PA, sedentary behavior, sleep, diet, and QoL as a result of the pandemic remains largely unexplored. The present study was designed to describe the patterns of Chinese adult PA, sedentary behavior, sleep, diet, and QoL under the COVID-19 pandemic, as well as the impact of the pandemic on their lifestyle behaviors and QoL compared with those outcomes before the pandemic.

Previous studies have suggested that PA and diet are critical components of healthy lifestyle behaviors, linking not only sleep quality but also the QoL [6,7,8,9,10]. Evidence has also indicated the critical role of sleep quality in mediating the effect of PA and/or diet on maintaining good health [11,12,13]. Gothe et al. proposed that a novel sleep and QoL model is useful to design new health interventions by targeting PA to promote sleep quality, which, in turn, influences the QoL [14]. However, given the massive impact of COVID-19 on all aspects of society, little understanding exists about how adult healthy behaviors, sleep, and the health-related QoL are related under the extremely restricted conditions in different regions of China. On the one hand, decreases in PA and increases in sedentary behavior (SB) could have negative impacts on the health-related QoL, sleep quality could mediate the relationships between healthy lifestyle behaviors (PA and food consumption) and QoL, and a bidirectional relationship may exist between these lifestyle factors and psychological health [15]. On the other hand, staying at home may also exert a positive influence on individuals′ eating behaviors while they stay with all family members and care more about their eating quality, in addition to being more relaxed from their daily busy work schedule. Thus, it is urgent to investigate whether sleep mediates the relationships among PA, diet, and QoL in Chinese adults during the pandemic.

Based on previous studies, we hypothesized the following: (1) Chinese adult PA would decrease while SB would increase, and dietary intake would become healthier during the pandemic, (2) the impact of the COVID-19 pandemic on human lifestyle behaviors and QoL would be mixed due to Chinese culture, and (3) sleep would mediate the relationships among PA, diet, and QoL in Chinese adults. Therefore, this study aimed to explore how COVID-19 is affecting the health-related QoL among Chinese adults. Specifically, we aimed to: (1) describe the patterns of Chinese adult PA, SB, food consumption, sleep, and QoL during the high peak of the lockdown from February to April 2020, (2) assess how healthy behaviors have been changed compared with those before the lockdowns, and (3) investigate the associations of PA, SB, and food consumption with each domain of the QoL (physical, social, psychological, and environmental domains) and the overall QoL, as well as whether those relationships were mediated by sleep quality during the lockdown. This study may provide useful and timely information to develop new action plans for physical education and public health services if another outbreak occurs (e.g., the Beijing COVID-19 outbreak in June 2020), as well as how to adopt a future “new normal” after the COVID-19 pandemic.

## 2. Methods

### 2.1. Study Design and Participants

#### 2.1.1. Participants

The study utilized a cross-sectional design with a convenience sample. The inclusion criteria were participants who: (1) were aged older than 18 years, (2) could read and understand the Chinese language and the purpose of the survey, and (3) had no diagnosed physical or mental disability. Eligible participants were asked about their physical and mental conditions before and during the COVID-19 isolation. The duration of the home isolation was, on average, 78 days (56–95 days), and the survey period ranged from 23 March to 26 April 2020. Originally, we anticipated obtaining 3000 samples for this survey. Finally, we successfully recruited 2289 participants from 34 provinces across China, yielding a response rate of 76.3%. 

#### 2.1.2. Study Design

We collected data on PA, SB, food consumption, sleep quality, and QoL from Chinese adults through commercial online survey platforms (i.e., WenJuanXing and WeChat). First, the participants′ demographic and socioeconomic information was collected (i.e., education, occupation, and physical and mental conditions). Additionally, the survey inquired about lifestyle behaviors, such as the levels of PA, SB, food consumption, smoking, drinking, sleep quality, and QoL. The internal reliability of the questionnaires was assessed using a confirmatory factor analysis.

The study was performed in accordance with the Declaration of Helsinki and was approved by the local ethics committee of the Exercise, Health and Technology Centre, Department of Physical Education at Shanghai Jiao Tong University, China (ML2019052). Informed consent was obtained from each participant before the beginning of the survey. The participants were volunteers without a monetary incentive, and they were informed about the use of their information.

### 2.2. Measurements

#### 2.2.1. Physical Activity

The PA level was assessed using the modified International Physical Activity Questionnaire Short Form (IPAQ-SF, Chinese version, which was translated by Qu [16]) [17]. The IPAQ-SF required participants to recall their PA engagement within the last seven days. The items were modified relative to prior to and during COVID-19 isolation (i.e., what is your vigorous PA level during the COVID-19 pandemic?) and activity types including typical traditional Chinese exercise; for example, “what was the frequency and duration spent on moderate PA (e.g., jogging, Tai Chi, and dancing) and low PA (e.g., yoga, Chinese traditional stretch exercise, and Baduanjin)” The guidelines for data processing and the analysis of the IPAQ-SF were followed [18]. The SB indicated the sitting and lying down hours spent on activities such as using a phone or computer or watching TV. The Cronbach′s alpha of the IPAQ-SF was 0.76. The IPAQ-SF has also shown acceptable measurement properties with Chinese populations [19].

#### 2.2.2. Food Consumption

The questionnaire for food consumption was adopted from the online nutritional survey of Guangdong Nutrition Society and Sun Yat-sen University with permission [20]. The consumption of different foodstuffs was collected during the isolation, and it was compared with that before isolation and included the weekly frequency and the total amount of consumption of whole grain products (e.g., rice, steamed buns, and steamed stuffed buns), meat products (e.g., pork, beef, fish, poultry, and eggs), dairy products (e.g., milk and yogurt), vegetables and fruits, snacks, alcohol, water, and soft drinks. The Cronbach′s alpha of the instrument in this questionnaire was 0.68 in this study.

#### 2.2.3. Sleep

Sleep quality was assessed using the Pittsburgh Sleep Quality Index (PSQI) [21]. The PSQI is a 20-item scale with seven domains: sleep quality, sleep duration, sleep latency, habitual sleep efficiency, sleep disturbances, sleep drug use, and daytime impairments. However, in this study, we did not include sleep disturbances; therefore, the total score calculation only included 6 items. Each domain was scored from 0 to 3, and a summed global score was calculated (ranging from 0 to 18). A higher PSQI global score indicates a lower sleep quality [22]. The Cronbach′s alpha of the PSQI was 0.82 in this study.

#### 2.2.4. Quality of Life

The World Health Organization Quality of Life (QoL) questionnaire (WHOQOL-BREF) [23] was used to assess the QoL. The WHOQOL-BREF contains four domains: psychological health (6 items), physical health (7 items), social relations (3 items), and environment-related domain (8 items). Additionally, two extra items were related to the overall perception of the QoL (Q1) and perceived health satisfaction (Q2). The items were scored on a 1–5 Likert scale (1 = strongly disagree; 5  = strongly agree). A global score was summed according to the domains, with higher scores reflecting a higher QoL. We obtained permission to use the English/Chinese version of the WHOQOL-BREF, and its Cronbach′s alpha in the current study was 0.89.

### 2.3. Statistical Analyses

Continuous data were checked for normality using the Shapiro–Wilk′s W-test with IBM SPSS Statistics 24.0 for Windows (SPSS, Inc., Chicago, IL, USA). If these continuous data were not normally distributed, their natural logarithms were used for further analysis. For objective 1, descriptive statistics were used to present the data means and standard deviation (SD). For objective 2, a cross-table Chi-squared (*χ*_2_) analysis was used to assess the changes compared with those before the lockdown, and the results are presented as numbers (*n*) and proportions (%). Additionally, we compared differences between gender, occupation, education, and living regions in health behaviors and QoL by independent *t* tests and analyses of variance.

**Figure 1:**
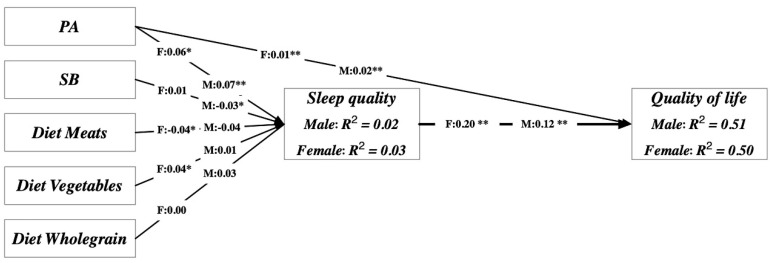
Scheme of path analysis. The model explained 50.9% (*r*_2_ = 0.509) and 2.4% (*r*_2_ = 0.024) of the variance in quality of life and sleep quality, respectively, in the male group and 49.6% (*r*_2_ = 0.496) and 2.8% (*r*_2_ = 0.028), respectively, in the female group.

Next, we used structural equation modeling (SEM) to test the third objective using Analysis of Moment Structure v25.0 [24]. A two-step process was involved. First, confirmatory factor analyses were conducted for the measurement model. The second step focused on testing all hypothesized relationships in the structural model (Figure 1). We used maximum likelihood estimation to evaluate the fit of the structural model to the data. Second, the criteria to assess the structural model were the same as those of the measurement model, using path significance or standardized regression estimates. An acceptable model fit for both the measurement and structural models was assessed using multiple indices, as described below. Finally, an invariance test and a bootstrap mediation analysis were conducted to discern the effects of gender and students vs. other adult differences on the mediating relationships among the study variables. The criteria for a good SEM model fit were applied as follows [25]: (1) a *χ*_2_/degree of freedom of less than 3; (2) a root mean square error of approximation (RMSEA) of less than 0.05; (3) a Tucker–Lewis index (TLI) and a comparative fit index (CFI), both greater than 0.90; and (4) a standardized root mean square residual (SRMR) of less than 0.05. The level of significance was set at *p* < 0.05 (two-tailed).

## 3. Results

The demographic and descriptive characteristics of the participants are shown in Table 1. The sample comprised 1176 (51.4%) men and 1113 (48.6%) women aged 18–81 years (Table 1). Approximately 70% of the participants were aged 30 years or younger. Most of the participants were from Shanghai, followed by the East China region, and most reported a college degree or higher education level. Sixty-one percent of the participants were students, 28% were office workers (including teachers, doctors, nurses, business office workers, and secretaries), 7.5% were retired or unemployed, and only 3% were industrial workers, farmers, and other types of physical workers. Most of the participants were engaged in work that was lightly physically demanding. Ninety percent of the participants reported not having chronic diseases (e.g., hypertension, diabetes, cardiovascular disease, hyperlipidemia, and cancer) and were nonsmokers. The male participants ate more whole grain and meat products than the female participants. Approximately 30% of the male participants drank alcohol, while only 8% of the female participants did.

### 3.1. Descriptive Patterns of Chinese Adult PA, SB, Food Consumption, Sleep, and QoL

**Figure 2:**
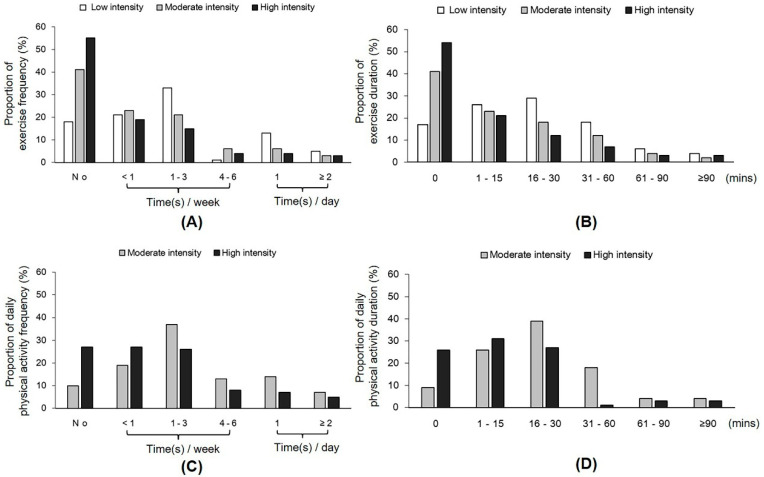
Proportion of people participating in physical activity (PA) during the home-isolation period. (**A**) Frequency of participation in different exercises. (**B**) Duration of participation in different exercises per time. (**C**) Frequency of participation in different daily physical activities. (**D**) Duration of participation in daily activities per time.

The participants’ PA behavior during the home-isolation period is presented in Figure 2 and Table 2. Most of the participants did not perform moderate-intensity PA (e.g., jogging, Tai Chi, and dancing; 40%) and vigorous moderate-intensity PA (e.g., rope jumping and weight training; 55%), and 18% did not perform light-intensity PA (e.g., slow walking, yoga, and the Chinese traditional stretch exercise Baduanjin) (Figure 2A). The duration of exercise per time is presented in Figure 2B. Few people reported performing vigorous PA with a short duration. Additionally, we assessed daily PA (such as housework, lifting light and heavy loads, and carrying a baby) during the isolation period (Figure 2C,D). Approximately 10% of adults did not perform any light PA, and approximately 26% did not perform vigorous PA. In terms of SB, 20–42% of the participants reported an increase. No significant differences were found in the energy expenditures of daily exercise and daily PA between men and women (Table 2).

**Figure 3:**
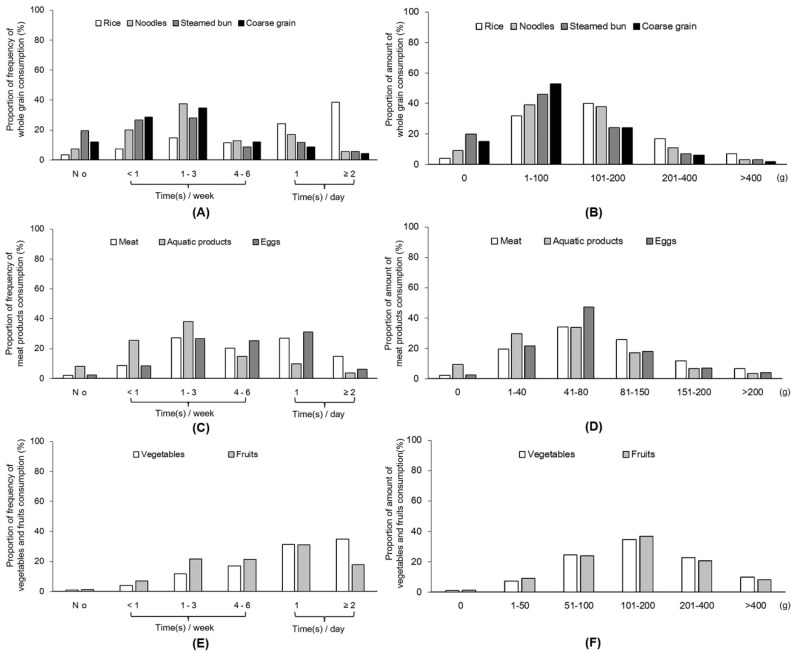
Proportion of dietary consumption frequency and daily amount during the home-isolation period. (**A**) Consumption frequency of whole grain. (**B**) Daily amount consumption of whole grain (**C**) Consumption frequency of meat products. (**D**) Daily amount consumption of meat products. (**E**) Consumption frequency of vegetable and fruit. (**F**) Daily amount consumption of vegetable and fruit.

The participants′ eating frequency and amount of food consumption are presented in Figure 3 and Table 2. Most of the adult food consumption fulfilled the recommendation of the dietary guidelines for Chinese residents (daily consumption of whole grain: 250–400 g; fish, poultry, meat, eggs, and other animal food sources: 120–200 g; vegetables: 300–500 g; fruits: 200–350 g; milk products: 100 g; and soy products: 50 g). Compared with women, men reported more frequently consuming whole grains, meats, milk products, and eggs but less frequently consuming vegetables and fruits (Table 2; *p* < 0.01 for all). Men also reported consuming higher amounts of whole grains, meat, milk products, and eggs. The overall amount of vegetables and fruits, did not differ between the genders.

Regarding sleep quality during home isolation, most of the participants rated their level of sleep quality as very good (75.2%), approximately 21.1% rated it as fairly good, 3.4% rated it as fairly bad, and only 0.3% rated it as very bad. Men reported significantly higher sleep quality than women (Table 2).

During home isolation, 65.3% of participants were satisfied with their QoL. The mean summed global score of the QoL was 188.9 (Table 2). Among the four domains, the highest mean scores were for the environmental domain and social relationships, while the lowest mean scores were for the physical domain. Only the environmental domain showed a significant gender difference (Table 2).

### 3.2. Change in Healthy Behaviors Compared with That before the Lockdown

**Figure 4:**
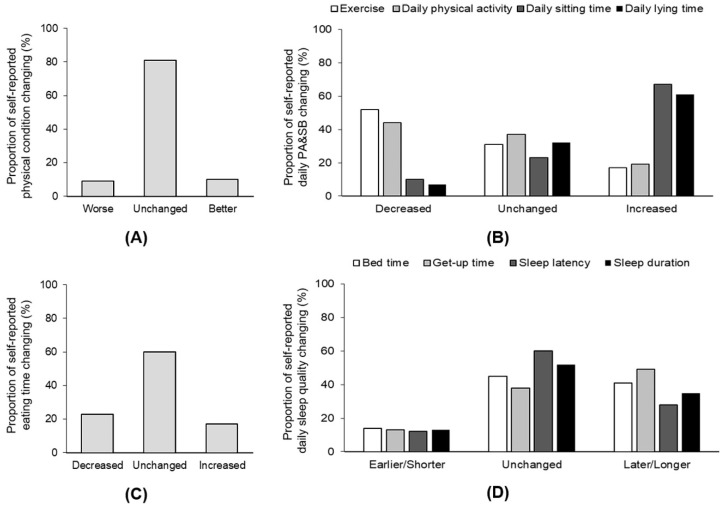
Proportion of self-perceived changes in physical condition and behaviors during the home-isolation period. (**A**) Change in physical condition. (**B**) Change in PA and sedentary behaviors. (**C**) Change in eating time. (**D**) Change in sleep quality.

Most of the participants reported that their physical condition remained at the same level (82%) as before home isolation, while approximately 9% of the participants reported their condition to be worse or better (Figure 4A). Fifty-two percent of the participants reported reduced levels of PA, while 17% increased their amount of exercise, 44% of the participants reduced their daily PA, and 19% increased their daily PA (Figure 4B). In addition, 67% of the participants increased their sitting time, and 61% increased their time spent lying down (Figure 4B).

Compared with their daily eating frequency before home isolation, 23.1% of the participants reduced their daily eating frequency, 17.3% increased their daily eating frequency, and 60% reported no changes during home isolation (Figure 4C). When asked whether they changed their appetite, 71.4% of the participants reported no changes. Notably, 23% changed their eating habits to be healthier. Regarding the different amounts of food consumption, more than 30% of participants consumed more vegetables, fruits, and milk products than before home isolation. Approximately 30% of participants reported an increased eating of snacks.

Forty-two percent of participants went to sleep late, and 49% woke up late in the morning compared with their sleeping behavior before isolation (Figure 4D). Approximately one-third of the participants reported longer times to fall asleep, and 35% reported sleeping longer (Figure 4D). Twenty percent of the participants reported feeling less energetic, while 11% reported higher energy levels.

### 3.3. Associations of PA, SB, and Food Consumption with QoL during the Lockdown

Table 3 displays the correlations between the variables analyzed by Pearson′s correlations (*r*). Specifically, PA was positively correlated with the QoL and negatively associated with SB in all samples (*p* < 0.01). SB was negatively associated with the QoL in the overall sample (*r* = −0.050; *p* < 0.01). Additionally, the consumption of whole grains, meat products, and vegetables and fruits were correlated with the overall QoL score in men (*p* < 0.001). However, only the consumption of vegetables and fruits was correlated with the overall QoL score in women (*r* = 0.117; *p* < 0.001) and was negatively with the physical domain (*r* = −0.06; *p* = 0.044).

The direct and indirect associations among the PA levels, SB, food consumptions, sleep quality, and QoL are presented in Table 4. The criteria for the validity and reliability of the measurement model were largely fulfilled, with a total composite reliability of 0.92 (criterion > 0.70) and an average variance extracted of 0.49 (criterion > 0.50). The model had an acceptable model fit (*χ*_2_/df = 780/288 = 2.7; *p* < 0.001; CFI = 0.87; TLI = 0.93; SRMR = 0.063; and RMSEA = 0.045), as indicated by the criteria of *χ*_2_/df < 3; CFI > 0.90; TLI > 0.90; SRMR < 0.08; and RMSEA < 0.05, respectively. The model explained 50.9% (*r*_2_ = 0.509) and 2.4% (*r*_2_ = 0.024) of the variance in the QoL and sleep quality, respectively, in men and 49.6% (*r*_2_ = 0.496) and 2.8% (*r*_2_ = 0.028), respectively, in women. We found that PA was positively associated with the QoL in both men (*p* < 0.01) and women (*p* < 0.01). Furthermore, significant direct relationships were found between PA and sleep quality (*p* < 0.001) and between sleep quality and QoL (*p* < 0.001) in men. Similarly, the relationships were significant in women between PA and sleep quality (*p* < 0.01) and between sleep quality and QoL (*p* < 0.001). The mediation effect of sleep quality for the relationship between PA and QoL was significant for the total effects (i.e., sum of the direct and indirect effects: Path Coefficients (β) = 0.03; Standard Error (SE) = 0.01; 95% Confidence Interval (CI) = 0.01–0.05; and *p* < 0.01).

However, SB showed no significant relationships with the QoL in both groups (men: *β* = 0.001; SE = 0.002; and *p* = 0.57; women: β = −0.002; SE = 0.004; and *p* = 0.58). No significant mediation effect of sleep for SB and QoL was found (β = 0.000; SE = 0.003; 95% CI = −0.006–0.007; and *p* = 0.93). Additionally, no direct relationships were found in men between the QoL and meat products (*p* > 0.05), whole grains (*p* > 0.05), and vegetables and fruits (*p* > 0.05), nor were any found between sleep quality and meat products (*p* > 0.05), whole grains (*p* > 0.05), and vegetables (*p* > 0.05). Similarly, significant direct relationships were not identified in women between diet and QoL, nor between sleep and diet. Therefore, no significant mediation effects of sleep quality were observed on the relationships between the QoL and the consumption of meat products, whole grains, and vegetables and fruits (See Table 4).

## 4. Discussion

In this study, more than two-thousand participants from 34 provinces in China provided reports on their health behaviors and QoL during the two-to-three-month COVID−19 home isolation. Home isolation had the most influence on the Chinese adult participation in daily exercise and PA. As expected, confining people staying at their homes led to an increase in a physically inactive lifestyle. However, adults were more aware of their eating and changed toward a healthier eating pattern. The overall satisfaction with their QoL was reasonable; however, the physical and psychological domains of the QoL were relatively low, and the social and environmental domain scores were higher than those in a previous study [26].

We found that more than 50% of the participants reduced their PA time and over 60% increased their sedentary time. The reason for the reduced PA time may have partly been due to small living quarters in densely populated urban cities, limiting the capacity of individuals to engage in PA. In this study, more than half of the participants were from Shanghai and the East China region, which have populations of 3823 people per square kilometer [27] and a living area of 36.6 square meters per person [28]. The participants living in these areas reported more decreases in terms of moderate-to-vigorous PA. This result urges us to develop a new type of PA to adapt to such limited living conditions.

Another possible explanation for the reduction in PA is increased screen times during lockdown. The combination of a low PA and high screen-time demonstrated the greatest negative impact on the QoL [29]. More than 60% of the participants reported increased hours sitting and lying down to play with mobile phones and computers and watch TV. Empirical studies have shown that a sedentary lifestyle is associated with many chronic diseases [30], immunity [31], and increased mortality [31]. Particularly, individuals older than 50 years who sat for more than seven hours a day had four times increased mortality after controlling for various confounders (gender, age, race, education level, smoking and drinking, body composition, diabetes, cardiovascular disease, cancer, stroke, and osteoarthritis) [32]. Sitting for a long period not only affects physical health but also poses a serious threat to mental health [33]. Additionally, most people enjoy exercising outdoors, which tends to elevate the mood, release pressure, and they are more likely to continue exercising in the future [34]. The lockdown also limited and weakened individuals’ exercise motivation because most of the venues and stadiums were closed and people were not familiar with workout routines for home training. Hence, the reduction in PA and increase in SB were mainly related to limited physical space and that people are not used to exercising at home during the COVID-19 pandemic in China.

Eating behaviors related to food choice and consumption are other important factors associated with sleep quality and QoL [35]. Before home isolation, most Chinese adults living in urban areas increased the frequency of eating outside due to the unwillingness to spend time in food preparation [32,36]. The 2011 “The Blue Book of the Catering Industry” annual report, a national survey comprising a population of 32,446, reported that approximately 70% of the respondents liked to eat outside the home. In addition to working meals, 50% of the consumers ate out one-to-three times a week, those who ate out four-to-six times accounted for 26.47%, and nearly 15% ate out every day [37]. The per capita food consumption increased from 5.7 Chinese yuan in 1980 to 2850 yuan in 2017, a 474-fold increase [38]. Eating outside has become routine in China due to improved living standards [38]. From the cultural perspective, although the proportion of Chinese eating in restaurants had risen sharply in recent years, most Chinese families still continue to the tradition of cooking their own meals at home because cooking is an important component of Chinese traditional culture. During home isolation, adults needed to cook and had time to make good decisions on food, and sharing meals with family also provided a positive impact through connection and communication [39]. Of note, there are numerous online shopping platforms in China. It was very convenient for people to buy fresh vegetables, fruits, and groceries online, even during the pandemic period. Meanwhile, China has a mature and developed logistics distribution system, so there was no real impact on individuals’ online shopping during home isolation with the express delivery services. In the current study, approximately 40% of the participants had increased their consumption of fruits and vegetables, particularly women. The consumption of vegetables and fruits was positively correlated with sleep quality in women, indicating that women were more likely to pay attention to their eating quality to control their body weight [40]. Additionally, both men and women had increased their water consumption and reduced soft drinks. The influence of home isolation indicated that it might have a positive impact on adult behaviors. The improvement of Chinese people′s eating habits during home-isolation is a novel finding of this study. It may shed light on individuals′ eating behavior and foster healthy eating behavior for those in other countries. However, the participants were not asked about their motivations for these changes; thus, we cannot draw reliable conclusions on this matter.

Interestingly, we found that most of the participants maintained their sleep quality. About 40–50% of the participants reported going to sleep and waking up later than before the COVID-19 outbreak. Only approximately 12–14% of the participants reported more difficulty falling asleep and a reduced sleep quality. However, 10–16% of the participants reported improved sleep quality during home isolation. This was likely related to people changing their routine schedule. For the Chinese, staying at home or working or studying from home for two-to-three months was in some way similar to a “long holiday.” Therefore, the COVID-19 outbreak afforded some people more time to relax to some extent. However, if home isolation is prolonged, the negative impact on individuals′ physical and mental health may be inevitable when new strategies are not enforced [41].

Using SEM, we found that sleep had a direct effect on the QoL and served as the mediator in the model. PA significantly predicted sleep quality and indirectly affected the QoL via sleep quality. This finding agreed with the results of a previous study [42]. However, the mediation effects of sleep quality were not observed among diet, SB, and QoL, likely largely due to the home-restricted dietary pattern and SB [43]. Thus, the results of the SEM suggest that daily PA participation is imperative in promoting the QoL among Chinese adults, regardless of SB.

To promote and maintain health, the American College of Sports Medicine and the American Heart Association have recommended that “all healthy adults aged 18–65 years need moderate-intensity aerobic (endurance) PA for a minimum of 30 min on five days each week or vigorous-intensity aerobic PA for a minimum of 20 min on three days each week” [44]. Clearly, during home isolation, most people did not meet the recommendation. However, those who reported higher PA participation had a better perception of their QoL and sleep quality. This finding agrees with that in a previous study [42,43,44,45,46]. Therefore, a national PA guideline in house confinement should be established that is suited for individuals and families to promote healthy lifestyles during crises.

This study had several strengths and limitations. A major strength of the study was the inclusion of a large sample of Chinese adults from many regions under the COVID-19 crisis to study their health behaviors and QoL through advanced statistical techniques. The present study data from questionnaires that have relatively good reliability and validity. However, the participants were mostly from Shanghai, and approximately 60% of them were college students, a finding that may have induced a bias of population representation in China. The SEM was based on previous knowledge to assess the direct and indirect associations among PA, SB, sleep, and QoL. Missing information, such as home living conditions and household members, may have played a role in explaining the outcomes. Additionally, 23% of the participants had already returned to work or study when the questionnaire was completed, a situation that may have affected their perceptions of health behaviors. Finally, due to the observational study design, cause–effect relationships among the study variables in this study could not be determined. A future study should adopt a longitudinal cohort design to explore the lifestyle health behaviors and QoL of Chinese adults.

To our best knowledge, this is the first study to investigate the status of PA, SB, diet, sleep, and QoL, as well as the associations among them, within a large Chinese adult sample during a global public health crisis. The conclusions drawn by this study contribute to the growing literature pool regarding PA promotion in a developing country population under unusual circumstances. COVID-19 is a new emerging situation that has dramatically impacted both individuals and their society. Our findings may provide useful and timely information in developing new action plans for physical education and public health services. In detail, the results may help inform healthcare professionals, practitioners, and educators on the status of Chinese adult lifestyle behaviors and health, in addition to offering innovative and effective PA programs at home during challenging circumstances such as a pandemic or heavy smog days. In future PA trials, it is necessary to use a randomized controlled design to examine the effectiveness of enjoyable and novel PA at-home programs given that most Chinese adults reported decreased PA during lockdown. Future home-based PA interventions could potentially focus on developing and maintaining physically active lifestyles for men and women alike. Finally, there is a need to investigate the effects of PA, SB, and sleep on cognitive functions [45] and mental health in adults in the future.

## 5. Conclusions

Our results indicate that the two-to-three-month home isolation during COVID-19 had mixed influences on Chinese adult health behaviors. Additionally, PA participation during containment measures was associated with better QoL and sleep quality levels, and sleep quality also served as the mediator in the model. People ate at home and focused more on their eating quality and patterns, which had positive influences on the QoL, particularly in women. However, the substantially reduced amount of moderate-to-vigorous PA warrants the identification of ways to enable and encourage people to engage in PA at home with limited space. Both government and communities should recommend practical guidance for adults to participate in PA with moderate-to-vigorous intensity that can be easily adopted at home, thus helping to maintain a healthy lifestyle in general and during a prolonged quarantine.

## Figures and Tables

**Table 1:** Participant demographic and descriptive characteristics by gender.

	Total (N = 2289)	Male (N = 1176)	Female (N = 1113)	*t*-Test
	Mean (SD)	Mean (SD)	Mean (SD)	*p*-Value
Age (yrs.)	27.5 ± 12.0	26.8 ± 11.9	28.2 ± 12.1	0.008
Height (cm)	169.6 ± 9.1	176.1 ± 6.6	162.7 ± 5.8	<0.001
Weight (kg)	64.3 ± 13.4	71.7 ± 12.7	56.6 ± 9.2	<0.001
Body Mass Index (BMI) (kg/m^2^)	22.3 ± 3.6	23.1 ± 3.6	21.4 ± 3.5	<0.001
Number (Proportion)	*n* (%)	*n* (%)	*n* (%)	Chi-Squared *p*-value
Region				0.902
Shanghai	676 (29.5)	352 (29.9)	324 (29.1)	
North China	278 (12)	135 (11.5)	143 (12.8)	
South China	491 (21.5)	253 (21.5)	238 (21.4)	
East China	611 (27.0)	315 (26.8)	296 (26.6)	
Macao, Hongkong, Taiwan	233 (10.0)	121 (10.3)	112 (10.1)	
Education				0.003
Under college	251 (11.0)	109 (9.3)	142 (3.8)	
University	1717 (75.0)	887 (75.4)	830 (74.6)	
Postgraduates and above	321 (14.0)	180 (15.3)	141 (12.7)	
Marital Status				<0.001
Single	1565 (68.0)	849 (72.2)	716 (64.3)	
Married	682 (30.0)	313 (26.6)	369 (33.2)	
Divorced or widowed	42 (2.0)	14 (1.2)	28 (2.5)	
Vocation				<0.001
Students	1408 (62.0)	769 (65.4)	639 (57.4)	
Others	881 (38.0)	407 (34.6)	474 (42.6)	
Work physical demanding				<0.001
Light physical activity	1513 (66.0)	760 (64.6)	753 (67.7)	
Moderate physical activity	299 (13.0)	120 (10.2)	179 (16.1)	
Vigorous physical activity	477 (21.0)	296 (25.2)	181 (16.3)	
Smoke				<0.001
No	2052 (90.0)	960 (81.6)	1092 (98.1)	
Yes	180 (8.0)	171 (14.5)	9 (0.8)	
Quit	57 (2.0)	45 (3.8)	12 (1.1)	
Drinking alcohol				<0.001
No	1811 (79.0)	794 (67.5)	1017 (91.4)	
Yes	434 (19.0)	349 (29.7)	85 (7.6)	
Quit	44 (2.0)	33 (2.8)	11 (1.0)	

**Table 2:** Comparison of physical activity, food consumption, sleep, and quality of life between men and women.

	Total (N = 2289)	Male (N = 1176)	Female (N = 1113)	*t*-Test
	Mean (SD)	Mean (SD)	Mean (SD)	*p*-Value
SB (hours/day)				
Sitting	7.4 ± 3.4	7.5 ± 3.3	7.3 ± 3.4	0.135
Lying down	9.2 ± 3.7	9.3 ± 4.2	9.1 ± 3.0	0.245
Exercise (Kcal/day)	287.8 ± 551.2	299.8 ± 607.6	275.1 ± 484.6	0.284
Daily activity (Kcal/day)	116.2 ± 150.8	110.4 ± 151.4	122.4 ± 149.9	0.057
Diets (g/day)				
Whole grain	246.1 ± 244.5	284.4 ± 282.9	205.5 ± 187.5	<0.001
Meat products	104.9 ± 115.9	120.8 ± 133.1	88.1 ± 91.6	<0.001
Vegetables and Fruits	131.6 ± 93.5	126.4 ± 94.8	137.1 ± 91.9	0.227
PSQI	3.8 ± 2.4	3.6 ± 2.4	4.0 ± 2.4	<0.001
Sleep quality	0.96 ± 0.6	0.94 ± 0.6	0.98 ± 0.6	0.096
Sleep latency	1.05 ± 0.9	0.98 ± 0.9	1.13 ± 0.9	<0.001
Sleep duration	0.32 ± 0.6	0.32 ± 0.6	0.31 ± 0.6	0.866
Habitual sleep efficiency	0.48 ± 0.9	0.41 ± 0.8	0.56 ± 0.9	<0.001
Use of sleep medications	0.07 ± 0.4	0.08 ± 0.4	0.07 ± 0.3	0.341
Daytime impairments	0.93 ± 0.9	0.88 ± 0.9	0.99 ± 0.9	0.002
QoL (score)				
Overall perception of QoL	3.6 ± 0.9	3.6 ± 0.9	3.6 ± 0.9	0.483
Satisfaction with health	3.6 ± 0.9	3.7 ± 0.9	3.6 ± 0.9	0.067
Physical	54.6 ± 13.2	54.7 ± 13.7	54.5 ± 12.7	0.736
Psychological	57.2 ± 13.9	56.9 ± 14.2	57.5 ± 13.6	0.315
Social relationships	69.8 ± 19.5	70.2 ± 20.6	69.4 ± 18.3	0.315
Environmental	69.8 ± 16.7	70.5 ± 17.0	69.1 ± 16.4	0.038
QoL (summed global score)	188.9 ± 40.4	189.2 ± 42.2	188.6 ± 38.4	0.749

Notes: Daily activity includes light activity (e.g., lifting light objects, cleaning, and scrubbing windows), vigorous daily activity (e.g., lifting heavy objects, dragging floors, and holding or carrying children). Whole grains include rice, steamed buns, steamed stuffed buns, noodles, and coarse grains. Meat products include pork, beef, fish, poultry, and eggs. Vegetables include leafy vegetables, wax gourds, tomatoes, and eggplant. Fruits include oranges, bananas, apples, cherries, strawberries, mangoes, peaches, pitayas, and pears. Abbreviations: SB = sedentary behavior; IPAQ = International Physical Activity Questionnaire Short Form; MET = metabolic equivalent; PSQI = Pittsburgh Sleep Quality Index; QoL = quality of life.

**Table 3:** Descriptive and correlational analyses among all outcome variables.

Variable	Total Sample (N = 2289)				Men (N = 1176)				Women (N = 1113)			
	1	2	3	4	1	2	3	4	1	2	3	4
1. Physical activity												
2. Sedentary behavior (Sitting and lying down)	−0.128 **				−0.099 **				−0.153 **			
3. Sleep (PSQI)	0.000	0.028			−0.008	0.038			0.001	0.024		
4. Quality of Life	0.212 **	−0.050 *	−0.023		0.207 **	−0.044	−0.042		0.219 **	−0.058	−0.001	

Note. ** Correlation is significant at the 0.01 level (2-tailed); * Correlation is significant at the 0.05 level (2-tailed). Abbreviations: PSQI = Pittsburgh Sleep Quality Index.

**Table 4:** Direct and indirect associations between physical activity levels, sedentary behavior (SB), diet, sleep quality, and the quality of life (QoL).

Path	Group	β	SE	*p*-Value	CI.L	CI.U
PA→QoL	Women	0.013	0.003	0.000 ***	0.006	0.020
Diet_wholegrain→QoL		−0.002	0.004	0.630	−0.009	0.006
Diet_meat→QoL		0.006	0.004	0.164	−0.002	0.013
Diet_VegeFru→QoL		0.000	0.004	0.947	−0.007	0.007
SB→QoL		−0.002	0.004	0.575	−0.009	0.005
Age→QoL		0.014	0.004	0.001 **	0.006	0.022
BMI→QoL		−0.006	0.004	0.085	−0.013	0.001
PA→Sleep		0.046	0.013	0.000 ***	0.021	0.071
Diet_wholegrain→Sleep		−0.025	0.017	0.153	−0.059	0.009
Diet_meat→Sleep		0.005	0.018	0.804	−0.031	0.040
Diet_VegeFru→Sleep		0.003	0.017	0.856	−0.030	0.036
SB→Sleep		0.002	0.016	0.926	−0.030	0.033
Age→Sleep		0.013	0.016	0.413	−0.019	0.045
BMI→Sleep		0.023	0.016	0.143	−0.008	0.055
Sleep→QoL		0.203	0.034	0.000 ***	0.136	0.269
PA→QoL	Men	0.014	0.005	0.003 **	0.005	0.024
Diet_wholegrain→QoL		−0.003	0.003	0.212	−0.009	0.002
Diet_meat→QoL		0.002	0.003	0.496	−0.004	0.008
Diet_VegeFru→QoL		0.002	0.003	0.539	−0.004	0.007
SB→QoL		0.001	0.002	0.570	−0.003	0.006
Age→QoL		0.016	0.004	0.000 ***	0.007	0.024
BMI→QoL		0.001	0.002	0.740	−0.004	0.006
PA→Sleep		0.073	0.024	0.003 **	0.025	0.121
Diet_wholegrain→Sleep		0.030	0.018	0.099	−0.006	0.066
Diet_meat→Sleep		−0.038	0.020	0.056	−0.077	0.001
Diet_VegeFru→Sleep		0.009	0.018	0.631	−0.027	0.044
SB→Sleep		−0.033	0.016	0.035 *	−0.064	−0.002
Age→Sleep		−0.018	0.017	0.265	−0.051	0.014
BMI→Sleep		0.016	0.017	0.363	−0.018	0.049
Sleep→QoL		0.128	0.031	0.000 ***	0.068	0.188
PA→Sleep→QoL	Mediation	0.009	0.003	0.002 **	0.003	0.015
Diet_wholegrain→Sleep→QoL		−0.005	0.004	0.163	−0.012	0.002
Diet_meat→Sleep→QoL		0.001	0.004	0.805	−0.006	0.008
Diet_VegeFru→Sleep→QoL		0.001	0.003	0.856	−0.006	0.007
SB→Sleep→QoL		0.000	0.003	0.926	−0.006	0.007
Age→Sleep→QoL		0.003	0.003	0.417	−0.004	0.009
BMI→Sleep→QoL		0.005	0.003	0.154	−0.002	0.011
Total effects		0.028	0.010	0.003	0.010	0.047

Abbreviations: PA = physical activity; QoL = quality of life; SB = sedentary behavior. β: Path coefficients; SE: Standard Error; CI.L: Lower limit of 95% Confidence interval; CI.U: Upper limit of 95% Confidence interval; * *p* < 0.05, ** *p* < 0.01, *** *p* < 0.001. “x→y”: path analysis examining the relationships between y (dependent variable) and x (independent variable).

## Abbreviations

The following abbreviations are used in this manuscript.

COVID-19	Coronavirus Disease 2019
PA	Physical Activity
QoL	Quality of Life
SB	Sedentary Behavior
IPAQ-SF	International Physical Activity Questionnaire Short Form
PSQI	Pittsburgh Sleep Quality Index
WHOQOL-BREF	World Health Organization on Quality of Life Brief Scale
SD	Standard Deviation
*χ*_2_	Cross-table Chi-squared
SEM	Structural Equation Modeling
RMSEA	Root Mean Square Error of Approximation
TLI	Tucker–Lewis Index
CFI	Comparative Fit Index
SRMR	Standardized Root Mean Square Residual
MET	Metabolic Equivalent
β	Path Coefficients
SE	Standard Error
CI.L	Lower Limit of 95% Confidence Interval
CI.U	Upper Limit of 95% Confidence Interval
*	*p* < 0.05
**	*p* < 0.01
***	*p* < 0.001
“x→y”	Path Analysis Examining the Relationships between y (dependent variable) and x (independent variable)
